# 

**Published:** 1916-06

**Authors:** 


### Deaths.

Dr. E. W. Munsey, of Corsicana, died May 2d.

Dr. J. W. Gann, of Huntington, died May 3d.

Dr. W. B. McGarity, of Belton, died May 20th.

Dr. Augustus Marable died at his home in Dallas recently.

Dr. J. F. Knott died at the home of his son in Dallas May 24th.

Dr. I. C. McCoy, a pioneer physician of Fort Worth, died April 29th.

William H. Bunge, a student of the freshman class of the University of Texas, died recently at the Sealy Hospital.

Dr. J. S. Hill, a well known and popular physician of Greenville, died May 21st after an illness of several months.

John A. Patton, President of the Chattanooga Medicine Company, Chattanooga, Tennessee, died at a local hospital April 26th after a short illness.

Dr. R. B. Turner, Chairman of the County Lunacy Board, Waco, was found May 2d in a chair with a bullet wound just behind the right ear and with a pistol in his hand in his office. He died soon after friends having offices nearby, and attracted by the shot, reached the room.

